# Scaling Up a Community-Based Exercise Program for Women in Difficult Life Situations in Germany—The BIG Project as a Case-Study

**DOI:** 10.3390/ijerph18189432

**Published:** 2021-09-07

**Authors:** Annika Herbert-Maul, Karim Abu-Omar, Anna Streber, Zsuzsanna Majzik, Jeanette Hefele, Stephanie Dobslaw, Hedi Werner, Alexandra Wolf, Anne K. Reimers

**Affiliations:** 1 Department of Sport Science and Sport, Friedrich-Alexander-University, 91058 Erlangen-Nuremberg, Germany; Karim.Abu-Omar@fau.de (K.A.-O.); Anne.Reimers@fau.de (A.K.R.); 2 Matrix GmbH & Co. KG, 91301 Forchheim, Germany; streber@matrix-gmbh.de (A.S.); majzik@matrix-gmbh.de (Z.M.); 3 Office for Sport and Health Promotion, City of Erlangen, 91054 Erlangen, Germany; jeanette.hefele@stadt.erlangen.de; 4 Health Department, District Administration of Regensburg, 93059 Regensburg, Germany; Stephanie.Dobslaw@landratsamt-regensburg.de; 5 Women’s Representative, Office for Social Services, City of Straubing, 94351 Straubing, Germany; Hedi.Werner@straubing.de; 6 Office for Children, Youth, Families and Integration, City of Bayreuth, 95445 Bayreuth, Germany; Alexandra.Wolf@stadt.bayreuth.de

**Keywords:** scaling up, health promotion, exercise, physical activity, socially disadvantaged groups, low socioeconomic status, ethnic minority, refugees

## Abstract

Scaling up community-based participatory research (CBPR) remains challenging. This case-study reports on how, and under which conditions, a CBPR project aiming at promoting exercise among socially disadvantaged women (BIG) scaled up at four project sites. As part of BIG, researchers support city administrations in implementing a participatory project to reach socially disadvantaged women for exercise. The case study was conducted in winter 2020 in southern Germany and is based on a co-creative process involving city administrators and researchers. Following Kohl and Cooley’s scaling up dimensions, scaling up BIG was investigated at the four sites using a mixed-method approach. Course registrations and offers were analysed, and qualitative interviews (*n* = 4) with administrative staff members were conducted and analysed using content analysis. The geographical coverage of exercise classes, the addressed groups, and the utilisation of participatory methods by city administrations are described. All four sites managed to scale-up project activities. Three of the four sites reported that further growth of the project was no longer possible due to limited resources. All sites attempted to reach a larger number of, and more diverse, women. One site managed to scale-up the use of participatory methods within the city administration. The following important facilitators for scaling up CBPR projects were reported: advertisements tailored to the needs of the addressed women, utilising participatory approaches, and equipping project coordinators with sufficient resources.

## 1. Introduction

To maximise sustainable health effects and public-health impact, effective interventions must be scaled up to other contexts or to a broader population [1]. The WHO defines scaling up as “deliberate efforts to increase the impact of successfully tested health interventions so as to benefit more people and to foster policy and program development on a lasting basis” [2]. In recent years, various scientific studies globally investigated the scaling of health interventions and identified factors that favour the scalability of interventions (e.g., proven effectiveness, engagement of stakeholders and partners outside the health sector, strong leadership, and secured financing in the long term) [3,4,5]. However, scaling up health promotion interventions is not a straightforward process; it is still described as challenging [3,6]. This holds true for interventions that follow a participatory methodology and attempt to reach disadvantaged groups. These interventions often address complex problems (e.g., physical inactivity) that are caused by multiple factors and are highly context-specific [6]. Such interventions cannot readily be transferred from one context to another, but rather need to be adapted to each context, and the population addressed [6,7]. This turns adapting a complex intervention into a tightrope walk between tailoring interventions to the context and, at the same time, maintaining its core elements to ensure effectiveness and sustainability [7,8].

Despite these challenges of scaling up, participatory approaches are of great interest for effective health promotion in communities. The community-based participatory research (CBPR) paradigm combines the process of taking actions and generating knowledge about the improvement of community health while equally engaging researchers and community members [9]. Through CBPR, it is possible to establish opportunities for participation and to achieve health-promoting effects on the individual (e.g., increase in self-efficacy) and structural (e.g., development of communal preventive services that are appropriate to the needs of the addressed groups) levels [9,10,11]. Furthermore, CBPR could reach groups with a low socioeconomic status because interventions are specifically adapted to the needs of the addressed groups [9,10,12,13,14]. This is a remarkable achievement because health-promotion interventions often fail to reach these groups [15,16,17,18]. However, physical-activity (PA) promotion among these groups is particularly important because disadvantaged groups have a higher incidence of noncommunicable diseases, and PA is a meaningful measure to prevent such diseases [19,20].

Socially disadvantaged women facing difficult life situations (e.g., due to a low socioeconomic status (SES) or difficulties because of belonging to ethnic minorities) are especially at risk of not exercising at a sufficient level. For example, women with a low SES are significantly less engaged in exercise than the population average [21,22,23]: a large-scale German survey found that 83% of women with a high SES reported being active in exercise in the last 3 months; for women with a low SES, this percentage was 50% [24].

The lower prevalence of exercise in socially disadvantaged women can be explained, among other things, by more limited opportunities to participate in exercise offers (e.g., due to high membership fees, limited availability, and offers that are culturally insensitive) [22]. Thus, there is a need for the development of adequate interventions to promote exercise among these population groups. In recent years, several CBPR projects to promote health among disadvantaged groups were developed and tested in study settings [10,12]. However, they were rarely scaled up, and the scientific evidence regarding the scaling up of CBPR is rather limited [6,25]. However, such evidence is relevant to facilitate the scale-up of CBPR to address the major challenge of physical inactivity among socially disadvantaged groups [4,5,6,26].

To narrow this research gap, the objective of the present case study is to generate knowledge on scaling up a CBPR intervention for exercise promotion among women in difficult life situations, e.g., women from ethnic minorities, unemployed women, and single mothers [27]. This case study describes scaling up BIG project (“Bewegung als Investition in Gesundheit” Movement as an Investment in Health) [27]. BIG aims at engaging the addressed women, and city administrations, to develop, implement, and scale-up low-barrier exercise offers in the community setting (low-barrier means that, e.g., there are minimal or no participation fees, no membership is required, childcare is offered, all instructors are female, and the offers take place close to where participating women live) [27]. Due to the long project duration and the implementation at several locations [28], the BIG project is particularly suitable as a case study for investigating the scale-up of CBPR. Applying the five different scaling up dimensions described by Kohl and Cooley [29], this article investigates the scaling up of the BIG project at four communities to answer the following research question: how and under which conditions do CBPR projects to promote exercise among socially disadvantaged groups scale-up on the local level?

## 2. Materials and Methods

The data and results of the study were generated in a co-creative process between researchers and the staff of city administrations who were in charge of developing and implementing low-barrier exercise offers jointly with women in difficult life situations. Co-creation means the collaboration of researchers and stakeholders from other sectors in order to collectively generate knowledge. Co-creation produces results that are highly applicable and influential in practice [30].

### 2.1. BIG as Case Study

The BIG project was developed in 2005 by researchers of the Friedrich-Alexander-University Erlangen-Nuremberg (FAU) and has, since then, been transferred to 17 communities [28,31]. In total, more than 800 women regularly take part in around 60 different BIG exercise offers [28]. The average project duration across all communities is approximately six years [28]. This long duration allows for studying how communities are managing to expand the exercise offers over time.

The aim of the BIG project is to implement low-barrier exercise offers to promote physical activity, and thus the health of women in difficult life situations [27]. Various barriers, such as high participation fees, necessity of membership, fear of stigmatization, language difficulties, a lack of culturally sensitive offers, or no featured childcare, make it difficult for these women to participate in existing offers.

The BIG project is based on a participatory approach [27]: Researchers in BIG collaborate with the staff of city administrations to enable them to set up local health-promoting structures (e.g., a local network of supporters, a project coordination office and funding stability) in the respective communities. The addressed women are integrated into these networks and are empowered to support the development and implementation of exercise offers. Thus, the implemented offers meet the needs of the women (e.g., low entrance fees, offered childcare, women-only hours at sport facilities) [27]. BIG achieves collaboration between the staff of city administrations and women in difficult life situations through cooperative planning. In several planning sessions, the participating women collaborate with local stakeholders such as political decision makers (e.g., mayors, sport-club chairs) and staff from the city administration. In the sessions, everybody contributes to the success of the planning process through their specific resources (such as contact with the addressed women or trainers, access to sporting facilities, and financial resources) [32]. As experts in their own behalf, the participating women provide a decisive perspective in order to develop need-based offerings and thus increase the acceptance of the offers among potential users. As an outcome of the cooperative planning, low-barrier exercise and PA offers are implemented (BIG offers mainly exercise courses such as swimming or Pilates and sometimes courses, e.g., to learn how to ride a bike in order to promote general PA. Unless otherwise stated, the term “exercise offers” is used below for both types of offers). Additionally, a local network of supporters is set up to ensure the long-term implementation and scaling up BIG at this site. The city administration assigns a local project coordinator with the task of sustaining the network and, if possible, scaling up the number of offers [28]. In the first years of project implementation, the collaboration between these coordinators or administrative staff and researchers from the FAU is very close; the communities receive extensive support in project implementation (e.g., consultation and seed funding). After one or two years, this support decreases, and the communities are more independent in the implementation of the project [28].

Table 1 contains a list of the different types of BIG offers that were implemented at the BIG communities.

The offers are advertised by peers—women who take part in the offers themselves. They inform other women in their social surroundings about BIG and accompany them to the offers. Previous studies on BIG found that BIG reaches the addressed women, and the exercise offers promote their physical and mental wellbeing [31]. Women who are involved in the cooperative planning expand their social network, their knowledge of organizational and political processes, and their skills in representing interests and experience self-efficacy [27]. Other stakeholders involved in BIG expand their social network and gain insights into the life situations and needs of the addressed women [33].

For two locations (Regensburg and Erlangen), sociodemographic data of the participating women were available (these data were not collected as standard at all sites, as this survey could have a stigmatizing effect on the women and limit the low-barrier nature of the courses). The age range of the participants was from 19 to 71 years, with an average age of 42.5 years. Approximately 60% of the women belong to ethnic minorities, and 42.6% of all women do not carry German citizenship. About 22% of the women receive some type of social welfare assistance.

### 2.2. Communities Represented in the Case Study

Four communities in Bavaria in Germany (Erlangen, Regensburg, Bayreuth, and Straubing) were selected for this case study to describe how BIG scaled up over time. At the time of data collection, the project duration at these sites was between 7 and 15 years (starting points: Erlangen, 2005; Regensburg, 2008; Bayreuth, 2011; Straubing, 2013). These communities were included in this study because the project implementation was particularly sustainable, and program fidelity was high (i.e., core project elements were preserved) at these sites. Thus, the expansion of the project could be observed over a long period. Other sites that had not implemented individual aspects of the project, e.g., the participatory method, or were not sustained over such a long duration, were excluded.

The number of inhabitants in the four communities was between 45,000 (Straubing) and 135,000 (Regensburg) [34]. In 2011, the proportion of people from ethnic minorities was at around 25% in all communities. According to the German Index of Socioeconomic Deprivation (GISD), all four communities were merely deprived (Erlangen and Regensburg belong to the least deprived quintile, and Bayreuth and Straubing to the second-least deprived) [35]. However, this does not mean that there is no socioeconomic deprivation at the neighbourhood level in these communities.

### 2.3. Data Collection and Analysis

The study applied the framework of Kohl and Cooley [29] for data collection and analysis to describe scaling up along five dimensions: breadth of coverage, depth of services, geographic coverage, client type, and problem definition (see Table 2 for a description). The study design (see Table 2) follows a mixed-method approach, i.e., quantitative and qualitative methods were combined. For the method description, we drew on the quality criteria formulated in the framework of Good Reporting of A Mixed Method Study (GRAMMS) from O’ Cathain et al. [36].

The mixed-method approach offers the possibility to comprehensively survey the different scaling dimensions by using the appropriate method in each case. The growth of the dimensions “breath of coverage” and “depth of services” can be described using the data from course registrations (quantitative data). The qualitative interviews can explain why course registrations developed that way by providing information on the other dimension and facilitators. The data from both methods were concurrently gathered and equally contribute to the main findings, as they each explore different scaling up dimensions.

Due to its participatory nature, data for this study were collected in collaboration between researchers and the administrative staff responsible for BIG in the four communities. At each site, the administrative staff involved in this study had been managing BIG for several years and accompanied and initiated the expansion of offers.

### 2.4. Qualitative Analyses of Interview Data

Qualitative data were collected by means of bilateral guideline interviews (*n* = 4) with the administrative staff member managing the BIG project in the communities. From Erlangen and Regensburg, two persons took part in the interviews. Accordingly, interviews were conducted with a total of *n* = 6 interviewees. Researchers prepared an interview guideline covering the five dimensions of the theoretical model. Additionally, the interviews touched upon potential facilitators and barriers for scaling up BIG at the site. The interview was transcribed verbatim and analysed using content analysis and the software f4. The transcripts were coded by a researcher using the scaling up dimensions and facilitators as codes. Afterwards, results of content analysis were discussed with the interviewees.

### 2.5. Analysis of Offers and Course Registrations

Researchers and administrative staff jointly analysed course offers and registrations (e.g., lists of participants, project reports, and flyers) from the four communities in order to analyse how many and what type of courses had been offered, and how many women had participated in these offers. For each year and site, registrations and offers were documented, to show their development over time and to compare the development across the different sites.

Following data collection, the researchers integrated the results analysis, summarised all relevant information, and shared these summaries with the administrative staff members. They checked, commented, and revised the summaries according to their perceptions.

### 2.6. Indicators

Number of registrations: As an indicator for the breadth of coverage, we analysed the number of registrations for the regular exercise and water sports courses because, at some sites and for some offers (e.g., other events, women-only pool hour), there was no documentation available. The number of course registrations is not equivalent to the total number of women participating, since women might take part in more than one exercise offer. At one site (Straubing), all courses are offered for 12 months. In Bayreuth, Regensburg, and Erlangen, most of the regular BIG courses run over a trimester; thus, there are three courses per year. To adjust the data, the number of courses offered in Straubing was multiplied by three.

Number of annual offers and diversity of offers: To analyse the number of annual offers, researchers and the administrative staff members screened documentation sheets, flyers, and project reports to collect data on annual exercise offers. They listed the BIG offers for each year of project implementation and categorised them into seven different types (offer types are described in Table 1).

Coverage of offers within the sites: regarding the geographical coverage of the offers within the sites, researchers and administrative staff members analysed if and to what extent the distribution of offers within the community evolved.

Diversity of addressed group: the researchers considered the diversity of the addressed groups by analysing if and how the addressed groups changed over the years, and which subgroups were specially addressed.

Usage of BIG methods for other problems or projects: researchers and administrative staff members reflected on whether the methods of BIG (cooperative planning, participatory approach, and peer approach) were implemented in other sections or projects, or extended to new problems (e.g., health promotion among long-term unemployed persons).

## 3. Results

**Figure 1:**
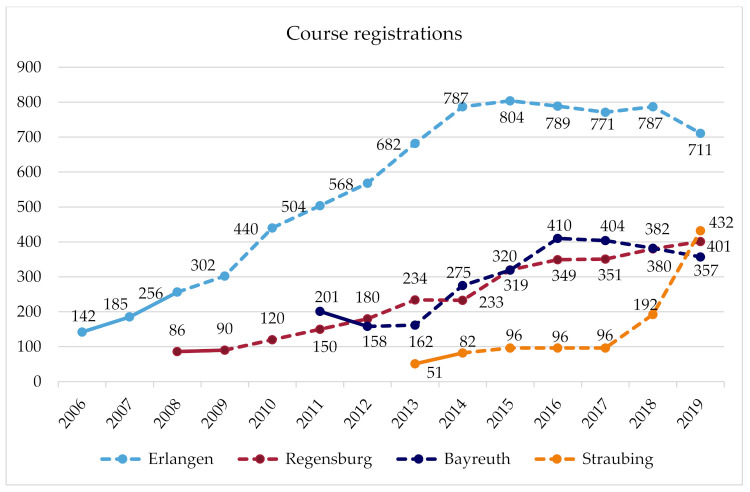
Number of women registering for regular physical activity course offers per year in four communities in Germany.

**Figure 2:**
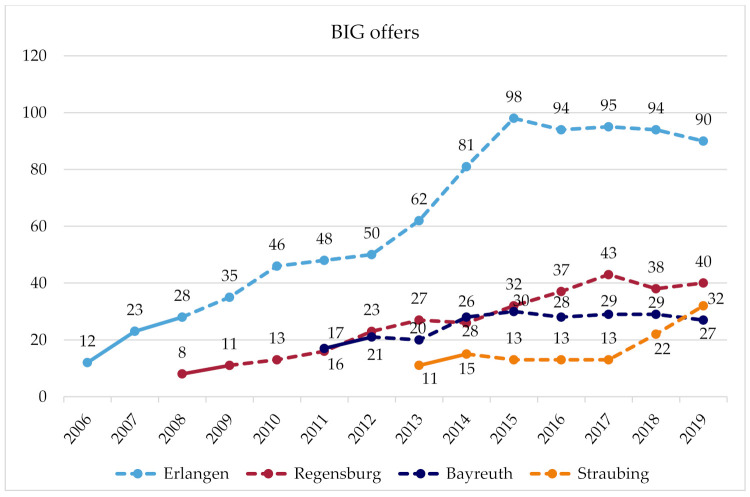
Number of BIG offers (all offer types) per year in four communities in Germany. Solid lines mark the years when communities received extensive support from researchers during project implementation (e.g., consultation, seed funding (Bayreuth did not receive seed funding)).

Figure 1 and Figure 2 illustrate the number of course registrations and exercise offers per year for each site. The figures also mark the time in which the communities received comprehensive support by researchers, and the subsequent years when the communities implemented the project more on their own term.

Table 3 summarizes the main findings for each scaling up dimension and site.

### 3.1. Erlangen

(Breadth of coverage: course registrations) The number of course registrations in Erlangen grew strongly over the years, and has plateaued since 2014. At around this time, further growth was no longer possible due to the limited financial resources and time of the administrative staff. In the following years, the number of registrations and courses settled at a level that is manageable within the framework of the given financial and time resources. In the last year, the focus shifted from reaching more women to instead maintaining the quality of offers and participation on a high level and paving the way for long-term BIG participants to take part in regular sports activities outside of the BIG courses, so that course capacities can be available to reach and include new disadvantaged women that have not participated in BIG so far. For reaching new participants, word-of-mouth advertisement is essential. The promotion of BIG offers is intentionally driven by the personal contact of peers and the administrative staff to the addressed women, but advertising also takes on a life of its own as participants bring their friends to the offers. (Depth of services: BIG offers) Every six months, a planning meeting takes place during which the women and administrative staff jointly plan a broad range of BIG offers for the next trimester. In addition to many exercise and water courses, bicycle courses, various irregular PA offers, and other events, such as meetings for social exchange, take place every year. In addition, a women-only pool hour and a train-the-trainer program on a yearly basis have been offered since 2006. The strong growth in offers between 2013 and 2015 is because offers for long-term unemployed women initially organised by a cooperation partner were integrated into the BIG project, as this cooperation partner could not continue these offers. (Client type: addressees) Initially, BIG addressed all women in difficult life situations. Later, a needs assessment was conducted to identify and subsequently to advertise the BIG offers to underserved groups, such as Russian-speaking women, single mothers, and long-term unemployed women, or women with an African or Asian migration background. Through network partners working at institutions for people with disabilities, BIG, today, additionally aims at reaching women with disabilities or experiences of trauma. To some extent, the new collaborations, addressing new target groups and recruiting new instructors, were implemented by the new coordinator, who has been managing BIG in Erlangen since 2019. (Geographical coverage) Offers were gradually created in an increasing number of city districts and facilities (e.g., rooms of community district centres), a development that is not yet complete. Currently, there are efforts to generate new offers in the east of Erlangen to reach the many women who live there and are so-far underserved. As women-only pool hours are rare, even women from outside of Erlangen make the trip to take part in this offer. (Problem definition: method scaling) In Erlangen, BIG significantly contributed to adopting the method of cooperative planning in various departments of the city administration. The BIG coordinator held training courses on how to implement participation and carried out participatory processes in other sectors. At the political level, the benefit of and the need for participation are today accepted. In addition, using the example of BIG, practitioners recognise the importance of outreach work and empowerment instead of solicitude.

### 3.2. Regensburg

The transfer project of BIG in Regensburg is called “FIT”. FIT is de-centrally structured as a number of social institutions (e.g., communal family centres and Campus Asyl), which are located in socially deprived urban districts, and independently plan and carry out FIT offers. The health department is responsible for the management and coordination of the network of these cooperation partners. Communal and regional agencies (the city and county of Regensburg, and the administrative district) assume the financial deficit of FIT, caused by the low course fees that do not cover the costs for facilities and trainers. (Breadth of coverage: Course registrations) The number of course registrations in Regensburg has been continuously growing. Many women initially come to the social institutions for other issues (e.g., cultural offers, childcare); once there, they hear about FIT, and they participate in the courses. Therefore, the recruitment of new participants is almost exclusively through word of mouth in the social institutions and in the social networks of participants. (Depth of services: FIT offers) Over the years, the number of cooperation partners offering FIT courses, and thereby the number of offers, has grown. Regular courses, bicycle courses, and a women-only pool hour are offered, and other irregular offers or events occasionally take place. In addition, a train-the-trainer program was offered in the past. Regarding the planning of offerings, participants are welcome to communicate their interests and needs. However, their participation is difficult, as most participants are solely interested in participating in the exercise offers but not in planning meetings. A further expansion of the offers is challenging due to the limited financial resources. Soon, additional funding will be applied for from a health insurance company to expand existing offers. Therefore, a needs assessment will be conducted to investigate which groups are underserved so far and how offers should be advertised (e.g., name of the offers, where to place advertisements) to reach these women. (Client type: Addressees) Nevertheless, FIT already reaches a diverse group of women (e.g., women from ethnic minorities, single mothers, older women, and especially those women who live in the vicinity of the social institutions). As a result of cooperation with a new refugee initiative (Campus Asyl) and as a result of the influx of refugees from 2015 onwards, FIT increasingly targeted and reached refugees. (Geographical coverage) Since the start of FIT, new cooperation partners in new city districts occasionally joined the FIT network and implemented offerings. Therefore, FIT offers are already distributed over a large area and cover several socially disadvantaged districts. In addition, efforts are underway to use the rooms of a new local meeting centre, which is located in a district where there is demand for offerings but where FIT has not been able to be established due to a lack of facility capacity. (Problem definition: method scaling) In Regensburg, many actors from different sectors use participatory methods, and they have recognised their benefits. However, this development commenced before the implementation of FIT.

### 3.3. Bayreuth

(Breadth of coverage: Course registrations) The initial high numbers of course registrations decreased in the second year because of problems with the rented premises, and misunderstandings with their owners (e.g., male janitors entered the room during the course or events took place in the room that had not been announced beforehand). As a result, some Muslim women in particular stopped attending the courses. This problem could only be solved after several arrangements had been made. In the following years, an increasing number of new addressee groups were reached, and the number of registrations increased again. Due to administrative reconstructions and the retirement of an administrative head who extensively supported BIG, the number of registrations slightly declined since 2016. (Depth of services: BIG Offers) Between 2011 and 2014, the number of offers per year grew, and there have continuously been almost 30 offers per year since then. A further expansion of offers is currently being prevented by limited facility capacities and a lack of trainers. The offers mainly consist of exercise and swimming courses; the types of courses depend on the interests of the women. Occasionally, bicycle courses, martial-arts courses, and other events are offered (Client type: addressees) While mainly Muslim women from Bayreuth initially took part in BIG, their proportion later declined because of the above-described problems. Instead, an increasing number of single mothers, women with a Russian migration background, and women from rural regions were reached, because word of mouth increased the awareness of the project among these women. In addition, since 2015, the offers increasingly opened and targeted to asylum seekers. (Geographical expansion) All exercise offers take place in the communal youth centre, and the swimming offers take place in the municipal pool or a school swimming pool. All facilities are centrally located in the city and are well-connected in terms of public-transport infrastructure. (Problem definition: Method scaling) It is not known whether BIG’s methods are used in other areas.

### 3.4. Straubing

(Breadth of coverage: Course registrations) In the first three years, the number of course registrations grew; it then remained constant for several years. Since 2018, there has been a strong increase in course registrations due to new courses for women from different cultural backgrounds, which arose from the new cooperation with a church congregation. (Depth of services: BIG offers) At the beginning, a very broad range of courses were planned together with the women. Due to a lack of registration for new trendy sports (e.g., Zumba), some courses did not come about. As a result, the range of courses was reduced to highly demanded sports. For two years, there was a women-only pool hour, which was initially busy. However, the swimming pool was located outside the city centre, so a transport service was organised for the women. Great effort was required to carry out this offer and when fewer women attended the courses, the offer could not be continued. In addition, discounted entrance fees for a fitness studio and a boxing studio have been offered since 2013. In 2018, a new offer was established due to the new cooperation with a church congregation, combining an exercise course and an integration course. Following the exercise course, the participants gather to talk and learn about language and culture. (Client type: Addressees) Due to this new offer, an increasing number of women from different cultures (including refugees and women with a migrant background) are targeted and reached. However, even before that, the group was heterogeneous (e.g., women with difficult family circumstances or low incomes, and single mothers were reached). (Geographical coverage) While the offers initially were de-centrally implemented, today they mostly take place in a socially deprived district in the south of Straubing, in the rooms of a local meeting place and a church congregation. These locations are particularly suitable, as they are close to where many addressed women live and are well-known in public. An extension of the BIG offer to the east of Straubing is planned. (Problem definition: Method scaling) BIG had a door-opening effect in Straubing. The range of activities has created relationships and trust between the women and the coordinators and trainers of BIG. Through these relationships, it became possible to successfully offer integration courses, and to openly talk about integration and culture with women from diverse cultural backgrounds.

## 4. Discussion

This case study provides valuable insights into the process of scaling up exercise offers for women in difficult life situations in four communities in Bavaria, Germany. In general, it can be seen as a success that the project grew at the four sites despite decreased support from researchers after the initial funding to set up the project ceased [6,37].

### 4.1. Communities Scale BIG on Their Own Terms

Strikingly, we were able to document how context-bound the BIG project was scaled at the four communities. Taking the breadth of coverage as an example, in the community of Erlangen, attendance to BIG courses showed quite a linear upward trend for eight years before reaching saturation. Conversely, in the community of Straubing, course attendance did not grow at all for four years before lately entering a seemingly exponential growth curve. Markedly, at three of the four sites, the number of offers (depth of services) has reached capacity due to limited resources (time, finances, facilities, and trainers). Therefore, two of those communities (Erlangen and Bayreuth) currently do not aspire a further scaling up of offers.

At all four sites, there have been efforts to reach new subgroups (client type). To some extent, these subgroups were deliberately selected because they were underserved up to that point or administrative staff recognised a particular need for measures among this group (e.g., refugees after the influx of refugees in 2015). In addition, efforts for special subgroups resulted from new collaborations with partners whose work targeted specific groups. However, the BIG approach was not extended to a very different client type (e.g., men in difficult life situations) or new problems (problem definition, e.g., other preventive services such as quitting smoking). Only the administrative staff of one site reported that, due to the implementation of BIG and on the basis of this good-practice example, the participatory approach and the method of cooperative planning are now used in diverse sectors of local government. While participatory approaches are increasingly used at the other sites, this development might not be associated with the implementation of BIG.

In addition, the multidimensional nature of scaling up stood out. So far, our perception of scaling up has been relatively narrow (i.e., we primary concentrated on the number of participants and offers (breadth of coverage and depth of services) and omitted other scaling up dimensions). However, at one site, rather unexpectedly, methods also scaled up to other sectors and problems.

Apart from the five dimensions described in the model, the awareness of the people involved also changed. In many cases, BIG created a positive attitude towards exercise and PA among the addressed women. Some of them became multipliers for PA, for example, they motivate people from their social surroundings to be physically active or take part in trainer’s education and afterwards carry out BIG offers themselves. Communal stakeholders admitted the necessity of health promotion among women in difficult life situations, recognised the benefits of BIG, and are well-disposed towards the project. This awareness, support, and participation of key actors are significant for increasing the impact of the projects [3,4,5]. Such broader social impacts of health-promotion projects are important, but hard to study (e.g., because the social impact can occur in various ways and years later, and causality between an intervention and its social impact is often not linear, and is influenced by many factors) [38].

### 4.2. Facilitators for Scaling Up

Our reflection as part of the qualitative data collection was able to identify important conditions to scale-up CBPR projects: (1) Appropriate advertisement is essential to increase attendance and to reach the addressed group. In addition, other studies found that peer approaches and word of mouth contacts were the most effective recruitment strategies in reaching disadvantaged groups [17,39]. (2) Participation of the addressed group is important for the process of scaling up [3,26]. Only through the engagement of the addressed persons (as experts on their own behalf) in planning and implementation can interventions meet their needs and interests, which is a prerequisite to their attendance. (3) Support of the project from politicians and administrative employees (e.g., financial resources or access to facilities) is essential for the project’s sustainability and scale-up. The review of Milat et al. (3), and the research of Lee et al. (4), and Nguyen et al. [40] also stressed the importance of strong advocacy and engagement of stakeholders, and long-term funding as key success factors for scaling up. (4) The personal capacities of the project coordinator are essential for scaling up. These capacities are, for example, time, authorisation to act, and the competence to network with the addressed women and to engage stakeholders in a participatory process. Prior research also emphasised the relevance of a strong leadership [3,6,29]. (5) The process of scaling up participatory projects for health promotion, so that the project can unfold its full public health impact, takes time. Even after years of project implementation, growth can continue. Correspondingly, other authors reported that the scaling up process can take upwards of 15 years [29], and effects of interventions only occur years after implementation [26,41].

### 4.3. Implications for Scaling Up CBPR

This study was conducted in Germany, and thus in a rather wealthy country. However, studies conducted in low- and middle-income countries reached similar conclusions regarding the facilitating factors of scaling up health interventions [5]. It is therefore possible that the results of this study are applicable to other countries around the world.

This study shows that scaling CBPR is challenging but possible. Our results indicate that, beyond the number of people reached by a CBPR intervention, it can be relevant to also explore how other project dimensions, such as the utilisation of participatory methods, are scaled. Particularly for CBPR projects that target policy and environmental changes, it is highly relevant to examine the broader social impacts achieved by such projects. Since scientific evidence on these social impacts is rather scarce, future studies on this topic could provide valuable results [38].

In addition, we were able to identify facilitating factors (e.g., appropriate advertisement and political support) that have been confirmed by other research and should be considered when scaling. Future studies examining barriers to this process would be additionally helpful.

Importantly, our results indicate that the CBPR interventions scaled differently at every project site. As such, in order to improve our understanding of how CBPR projects scale, mixed-method approaches that combine ethnographic data with documentations of project participation seem to be useful.

### 4.4. Strengths and Limitations

With its results, this case study contributes to the currently limited scientific evidence on scaling up CBPR and its facilitating factors by providing in-depth information from four communities on the basis of mixed-method analysis. It provides structured analysis of five scaling up dimensions derived from theory. The mixed-method approach proved to be very valuable in this study, as the quantitative and qualitative data complemented each other well in exploring the five dimensions. A particular strength of this study is the long time period over which the scaling-up process was analysed. Due to its co-creative approach, this study generated results that are highly applicable in practice and can be of great interest for others intending to scale-up CBPR.

Apart from these strengths, we acknowledge some limitations of this study. First, we reanalysed documents such as registration lists to document the development of course and registration numbers over time. Further statistical analysis was, therefore, not possible. Potential course-registration data from the project sites might have been incomplete. Furthermore, our analysis focused on communities where BIG has been running for years and is deemed to be successful. As such, these case examples do not allow for inferences to all other communities where BIG is running. Moreover, all four sites belong to rather less deprived cities according to the GISD data. Although all four communities feature deprived areas within their community limits, these communities might have more available financial resources to support BIG compared to those of other communities.

The scaling up process that we described used only certain dimensions, as set out by Kohl and Cooley [29]. We might have missed other dimensions of growth that the model does not cover (e.g., breadth of contact to other organizations) and that could also be relevant. In addition, we barely investigated barriers that might impede scaling-up, and focused instead on the facilitators. Furthermore, we did not examine how these sites implemented the project, and instead focused narrowly on the number of offered courses and participant rates. However, how certain adaptions influenced the effectiveness, and thereby the scaling up process of BIG, is described elsewhere [28].

## 5. Conclusions

Scaling up health interventions is challenging. This holds particularly true for complex interventions such as CBPR projects that intend to promote health among disadvantaged groups. In this study, we examined how the staff of four city administrations were able to scale-up such a project on their own terms after the initial funding and support from research to set up the project ceased. We identified the following enabling factors for a successful scale-up: advertisements that meet the needs of the addressed persons, participatory approaches, the support of key stakeholders, competences to network, the authorisation to act of a project coordinator, and time, since the process of scaling up can drag on for years. More research on scaling up complex interventions like CBPR, e.g., on barriers to successful scale-up, could increase the impact of proven public-health interventions.

## Figures and Tables

**Table 1:** Descriptions and examples for different types of BIG offers that were implemented at the sites.

Type of BIG Offers		Description/Example
1	Regular exercise courses	Course on a mostly weekly basis, e.g., yoga, dance courses, back fitness, archery.
2	Regular water-sport courses	Course on a mostly weekly basis, e.g., swimming courses, aqua fitness
3	Bicycling courses	Courses to learn or advance abilities to ride a bike
4	Women-only hours	In public pools or gyms All attendees, lifeguards, and trainers are female
5	Irregular PA offers	Cycling or hiking day trips, climbing
6	Other events	Martial-arts courses, seminars on a healthy lifestyle, workshops, network meetings for social exchange.
7	Train the trainer program	Programme to train the addressed women to lead exercise courses.

**Table 2:** Study design (study dimension and description following Kohl and Cooley [29]).

Scaling up Dimension and Description	Indicator	Method and Analyses
Breadth of coverage (extending to more people in currently served categories and localities)	Number of registrations at regularly course offers	Analysis of course registration and offers, coproduction of results section
Depth of services (extending additional services to current clients)	Number of annual offers	
	Diversity of offers	Qualitative bilateral guideline interviews with administrative staff, coproduction of results section
Geographical coverage (extending to new sites)	Coverage of offers within the site	
Client type (extending to new categories of clients)	Diversity of addressed group	
Problem definition (extending current methods to new problems)	Usage of BIG methods for other problems or projects	

**Table 3:** Summary of main findings.

Scaling up Dimension	Sites				All
	Erlangen	Regensburg	Bayreuth	Straubing	
Breadth of coverage: course registrations (see Figure 1 and Figure 2)	Strong increase over several years; saturation since 2014 due to limited capacities (time, money).	Number of registrations is continuously growing.	Registration rates increased for five years until saturation due to limited resources (facilities, trainers).	No growth for a number of years; sudden fourfold growth due to a new collaboration	Each community has its own trajectory of growth; at one point, saturation was reached due to limited resources
Depth of services: BIG offers (see Figure 1 and Figure 2)	Broad course program, planned in collaboration with participants; saturation since 2014 due to limited capacities (time, money).	Over the years, the number of offers grew, but recently reached saturation due to limited financial resources.	Number of offers somewhat increased until it had reached saturation due to limited resources.	Number of courses remained constant over the years, and increased markedly due to a new collaboration.	Tendency to broaden offers until saturation is reached due to limited resources.
Client type: addressees	Women in difficult life situations. Shifting focus on differing underserved subgroups.	Women in difficult life situations. Since 2015, more refugees have been approached.	Women in difficult life situations. Shifting focus on underserved subgroups. Since 2015, more refugees have been approached.	Women in difficult life situations. Since 2015, more refugees and women from different cultures have been approached.	All women in difficult life situations, shifting focus on underserved groups.
Geographical coverage	Offers are spread within the community; increased geographical coverage over time.	Offers are spread within the community; increased geographical coverage over time.	Offers limited to city centre.	Offers limited to city centre; further expansion is planned.	No automatic increase in geographical coverage.
Problem definition: method scaling	Various departments of city administrations adopted participatory methods. Political decisions makers acknowledge the added value of these methods.	No scaling of participatory methodology.	No scaling of participatory methodology.	Established relationships to women were utilised by the city administration to plan other service offers.	No automatic adoption of BIG methodology, but potential for capacity building through BIG methods exists.

## Data Availability

The data generated and analysed in this study are available upon reasonable request from the corresponding author.
